# Successful Use of Tofacitinib in Scleroderma Arthropathy

**DOI:** 10.31138/mjr.34.2.266

**Published:** 2023-06-30

**Authors:** Prakashini MV, Debashis Maikap, Prasanta Padhan

**Affiliations:** Department of Clinical Immunology and Rheumatology, Kalinga Institute of Medical Sciences, KIIT University, Bhubaneswar, Odisha, India

**Keywords:** scleroderma arthropathy, rheumatoid arthritis, erosion, tofacitinib, systemic sclerosis

## Abstract

Musculoskeletal manifestations of systemic sclerosis (SSc) are frequent and may be one of the early manifestations of the disease. However, arthralgia, pain and stiffness without frank arthritis usually constitute the clinical picture, while overlap syndromes such as rheumatoid-like polyarthritis can dominate when the arthritis is erosive. Hereby, we report a case of primary SSc presenting as frank erosive arthritis involving small and large joints mimicking rheumatoid arthritis, unresponsive to methotrexate, which was successfully treated with tofacitinib.

## INTRODUCTION

Musculoskeletal manifestations are common in systemic sclerosis (SSc), and the presentation of joint involvement is quite variable, ranging from arthralgia to frank polyarthritis with or without tendinopathy.^1^ Articular involvement is an important determinant of disability and impaired quality of life in SSc. MSK pain syndromes in SSc include polyarthralgia/polyarthritis, tendonitis, bursitis, and overlapping rheumatoid arthritis (RA).

Joint symptoms have been noted in 12 to 66% of patients at the time of diagnosis and in 24 to 97% of patients at some time during the course of their illness.^2^ Histological evidence of synovitis has been found in up to 66 % of synovial biopsies from patients with SSc, but, clinically, arthralgia is considered to occur more frequently than frank arthritis.^3^ Regrettably, arthritis-specific outcome measures for SSc have not yet been thoroughly validated, and the treatment of arthritis in SSc has not yet been investigated as a primary endpoint in randomised controlled trials. Low dose corticosteroids, hydroxychloroquine and methotrexate are commonly used in the treatment of scleroderma related arthropathy. Biologics like B-cell-depleting therapies (rituximab), tocilizumab and TNF inhibitors have also been tried in many case series with variable results.^4^

There is recent evidence that the JAK/STAT signalling pathway is markedly activated in SSc patients. In genetic studies, STAT locus variants were shown to be strongly associated with SSc. Therefore, JAK/STAT signalling may have crucial role in the pathogenesis of SSc.^5^

Tofacitinib, which inhibits JAK1/JAK3, has displayed benefit in systemic lupus, (RA), psoriatic arthritis and ulcerative colitis. There is however, a dearth of information on the usage of JAK-inhibitors in SSc. When JAK/STAT activity is inhibited by Tofacitinib, it eliminates the key fibrotic responses in fibroblasts and stops mouse multiple organ fibrosis. These results were the first to show that tofacitinib treatment may be successful in delaying or reversing fibrosis in SSc patients with genetic evidence of increased JAK/STAT pathway activation in target organs.^6^

## CASE REPORT

**Figure 1.**
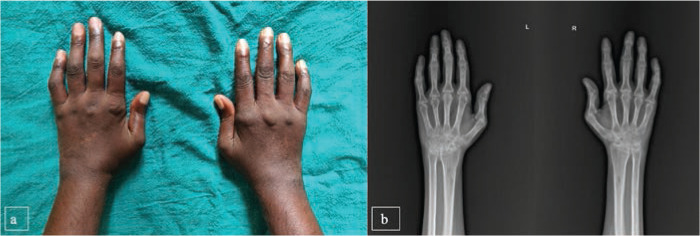
**(A)** bilateral sclerodactyly and Z-deformity of the thumbs with wrist swelling. **(B)** X-ray of both hands showing joint space narrowing at MCP, PIP, DIP with articular surface erosions and Z-deformity of the thumb.

A 28-year-old female presented with symmetric polyarthritis involving ankles, knees, metacarpophalangeal joint (MCP), proximal interphalangeal joint (PIP), DIP, elbows associated with significant early morning stiffness of 2 hours duration. On examination, she had Raynaud’s phenomenon, sclerodactyly (**Figure 1A**), restricted opening of the mouth and healed digital pits over the fingertips, tendon friction rubs over the right wrist, skin thickening of her fingers distal to metacarpophalangeal joints (MCP), on the thighs, legs, face and feet (Modified Rodnan Skin score, mRSS of 18), with swelling and tenderness of both ankles, knees, wrists, MCP and proximal inter-phalangeal (PIP) joints. Her complete blood count, renal and liver functions were normal. Acute phase reactants were raised (ESR- 45mm/1^st^ hour, CRP- 24mg/L) with a positive antinuclear antibody (ANA) and anti-Scl-70. Her Rheumatoid factor (RF) and anti-CCP were negative. X-ray the hands showed marginal erosions at MCP, PIP, DIP (features are described in the image **Figure 1B**). Her DAS28 score was 7.31. HRCT thorax and echo-cardiography were normal. A diagnosis of scleroderma arthropathy was made and weekly oral methotrexate 15mg with Prednisolone 7.5 mg daily were started. At 12 weeks’ follow-up, she had inadequate response to methotrexate, with her joint pains and swelling persisted with DAS28 score of 7.69. Tofacitinib, was given at a dose of 5mg twice daily after ruling out latent tuberculosis infection. At 4 weeks’ follow-up, her joint swelling and mobility improved significantly with DAS28 falling to 2.6 and normalization of acute phase reactants (ESR – 15mm/1^st^ hour and CRP – 4mg/L). However, there was no significant improvement in the modified Rodnan skin score at 12 weeks (mRSS-16).

## DISCUSSION

Articular manifestations may be the presenting feature in 24–96% of patients of SSc and are clinically manifested as arthralgia, flexion contractures and less frequently as true arthritis.^7,8^ Articular involvement in early SSc may resemble rheumatoid arthritis (RA) but is seldom erosive,^9^ with MCP and PIP joints being commonly affected. However, the presence of positive RF and anti-CCP neither correlates with the clinical nor radiographic pattern of arthritis in SSc^10^. According to the data from EUSTAR registry, patients with early synovitis were predicted to have a more severe course with diffuse skin involvement and this can serve as a potential prognostic factor in due course. Frequency of hand erosions was variable, between 5–40%.^11^ A recent cross-sectional study observed the presence of DIP joint erosions (15%) and DIP joint space narrowing (21%) in SSc patients compared to healthy controls suggesting that DIP changes might be specific of SSc.^12^ There is also recent evidence that JAK/STAT signalling has a role in the pathogenesis of SSc.^13^ A pilot study demonstrated that tofacitinib had a superior effect over methotrexate in terms of skin thickness and musculoskeletal manifestations in SSc, without an increase in adverse events.^14^ It has also been observed that involvement of the first carpometacarpal (CMC) join may be a distinct feature of SSc.^14^

In our case, erosive arthritis with diffuse synovial hyper-trophy was observed in the hands, including DIP, 1^st^ CMC, knees, and ankles. This case highlights two important aspects. First, the arthritis of systemic sclerosis can mimic diffuse erosive picture of rheumatoid arthritis, but with the involvement of DIP which may be relatively specific to SSc.^5^ Second, tofacitinib has emerged as a novel treatment option for erosive arthritis in SSc, which is refractory to methotrexate and low dose steroids.
